# Study on the lignin-derived *sp*^2^–*sp*^3^ hybrid hard carbon materials and the feasibility for industrial production

**DOI:** 10.1038/s41598-024-54190-x

**Published:** 2024-03-01

**Authors:** Si-Yu Long, Yan Qin, Jin-Lei Liu, Xue-Quan Xian, Ling-Qiang Zhou, Wen-Da Lv, Pei-Duo Tang, Qin-Yan Wang, Qi-Shi Du

**Affiliations:** 1 National Key Laboratory of Non-food Biomass Energy Technology, Guangxi Academy of Sciences, Nanning, 530007 Guangxi China; 2 Fujian Yuanfu Biomass Technology Co., Ltd., Jiangle, Sanming, 353300 Fujian China

**Keywords:** Biomass, Lignin, Hard carbon, Sodium ion battery, Graphene, Materials science, Chemistry, Materials chemistry

## Abstract

Hard carbon has been widely used in anode of lithium/sodium ion battery, electrode of supercapacitor, and carbon molecular sieve for CO_2_ capture and hydrogen storage. In this study the lignin derived hard carbon products are investigated, and the conclusions are abstracted as follows. (1) The lignin derived hard carbon products consist of microcrystal units of *sp*^2^ graphene fragments, jointed by *sp*^3^ carbon atoms and forming *sp*^2^–*sp*^3^ hybrid hard carbon family. (2) From the lignin precursors to the *sp*^2^–*sp*^3^ hybrid hard carbon products, most carbon atoms retain their original electron configurations (*sp*^2^ or *sp*^3^) and keep their composition in lignin. (3) The architectures of lignin-derived hard carbon materials are closely dependent on the forms of their lignin precursors, and could be preformed by different pretreatment techniques. (4) The carbonization of lignin precursors follows the mechanism “carbonization in situ and recombination nearby”. (5) Due to the high carbon ratio and abundant active functional groups in lignin, new activation techniques could be developed for control of pore size and pore volume. In general lignin is an excellent raw material for *sp*^2^–*sp*^3^ hybrid hard carbon products, a green and sustainable alternative resource for phenolic resin, and industrial production for lignin derived hard carbon products would be feasible.

## Introduction

Hard carbon is a type of carbon materials that cannot be graphitized under very high temperature even over 2500 °C^1,2^. On the other hand, at temperature around 1400 °C soft carbon could be converted to graphite crystal over 90%^3,4^. In recent decades hard carbon has found important applications in anode materials of lithium ion battery^5–7^ and sodium ion battery^8–12^, electrode materials of supercapacitor^13–16^, and carbon molecular sieves for gas separations^17^, such as H_2_/CH_4_ and N_2_/O_2_ separations^18–20^, and CO_2_ capture^21^, all are urgent needed materials for the solutions of energy crisis, climate change, environment and ecology protection^22–25^. The traditionally raw materials of hard carbon are several synthetic resins, such as epoxy resin, ion exchange resin, and phenolic resin^26–28^, all are manufactured from coal and petroleum. Seeking for alternative and sustainable feedstocks for hard carbon production is an urgent task.

Traditionally biomass had long been the precursors of various carbon products for hundreds and thousands years. So far many researchers still keep searching different biomass species for various carbon materials, such as eucalyptus wood, corncobs, peanut shells, waste apples, and orange peel^29–31^. On the other hand, more detailed study revealed that all biomass species consist of three components: cellulose, hemicellulose and lignin. The carbon products fabricated from the three components of biomass are quite different in structures and properties, also different from the carbon products directly produced from biomass species^32–35^. Among the three components of biomass, lignin is the second-abundant component, very different from the other two carbohydrate components (cellulose and hemicellulose) in chemistry and physics properties. It has been noted that a variety of high-value carbon materials can be made from lignin, such as low-cost carbon fiber^36,37^ and carbon black to strengthen tires^38^. Therefore, lignin is a green and sustainable resource with great potential to produce high-value carbon materials.

The natural occurrent lignin has been estimated to be around 0.5–0.6 billion tonst annually, as well as about 40–50 million tons of lignin from pulp and paper industry and 100,000–200,000 tons from cellulosic ethanol industry as a pollutant byproduc^39,40^. Therefore the most abound and available lignin resource is the pulp mills, where the cellulose are extracted from wood or grass feedstock for paper pulp, mean while lignin is discarded in black liquor. In black liquor lignin chemically combines with alkali (sodium hydroxide) through ester bonds and salt bonds, forming dissoluble alkali lignin. For high-quality hard carbon products, lignin has to be further extracted and purified from black liquor by means of acidification reaction or electrodialysis. This study is devoted to exploring technical and theoretical issues of lignin derived hard carbon, particularly the feasibility of its industrial production.

## Methodology and lignin materials

### Methodology

The lignin precursors and carbon products are measured and characterized using physical and chemical methods. The SEM (Scanning electron microscope) images are taken by using an instrument Hitachi S-3400. In order to obtain clearer SEM images, the samples are first coated by gold-beam. The crystal structures of lignin samples and derived carbon products are characterized by the HRTEM (High Resolution Transmission Electron Microscope). In this study the HRTEM images are taken by a commercial cororation (Tianhe (Shandong) Testing Technology Co, Ltd, https://www.keysci.com/) using Transmission Electron Microscope (Tecnai G2 F30). The Minimum Dose Sytem and other helpful tools are integrated into this instrument. In order to avoid samples to be damaged, when the HRTEM images of lignin samples are taken, the irradiation intensity and imaging conditions are carefully adjusted and selected. The chemical structures of lignin samples are identified by using FTIR (Fourier transform infrared spectroscopy) instrument (Thermo fisher Nicolet IS10). The crystal structures of lignin derived hard carbon materials are characterized by using laser Raman spectroscopy (Renishaw inVia Raman spectrometer with a laser wavelength of 532 nm). The specific surface area (SSA) and pore structure of active carbon is measured and characterized by using Automatic Volumetric Sorption Analyzer (Autosorb-1MP, Quantachrome) with BET (Brunauer, Emmett and Teller)^41^ and DFT (density functional theory)^42^ methods. The atomic composition and electron states on the surface of lignin precursous and derived carbon products are analyzed by using XPS (PHI Quantera II)^43,44^.

### Lignin raw materials

In this study the black liquor was provided from Yuanfu biomass technology Co. Ltd in Sanming, Fujian, where bamboo and sodium hydroxide were used as the feedstock for paper pulp production.

#### Lignin structure and characters

**Figure 1:**
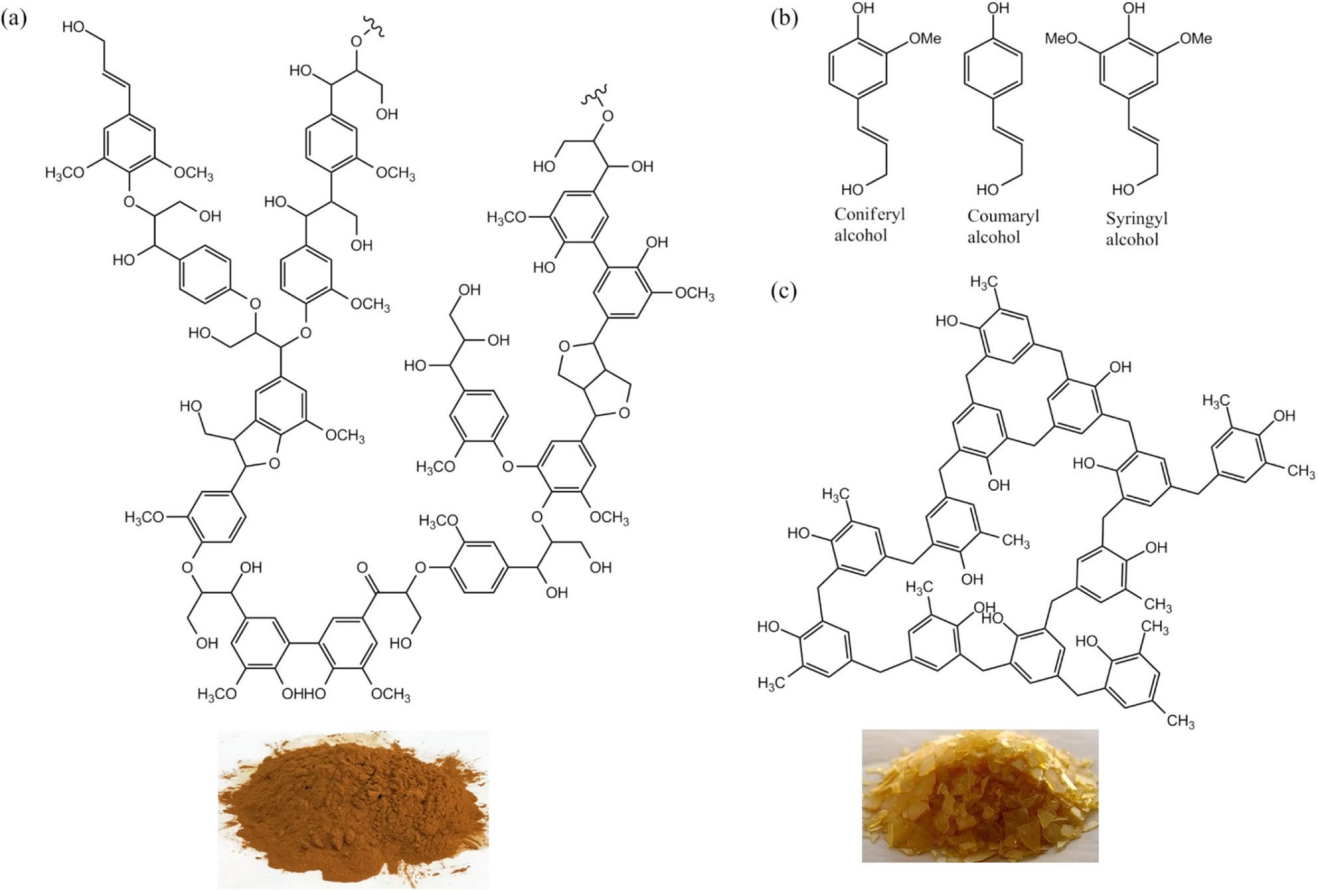
Molecular structures of lignin and contrast phenolic resin. (**a**) A molecular fragment of lignin. Lignin is the polymer of phenolic monomers, and has no definite molecular formula and no crystal structure. (**b**) Three phenolic monomers: coniferyl alcohol, coumaryl alcohol, and syringyl alcohol. (**c**) A molecular fragment of phenolic resin.

Lignin is a random polymer of three phenol monomers withyout definite chemical formula and has no fixed crystal structure. An approximate empirical chemical formula of lignin (C_31_H_34_O_11_)_n_ was proposed by Faix et al.^45^. Based on this formula of lignin the element composition is around carbon 63.4%, oxygen 30%, and hydrogen 5.9%. Figure 1a shows a structural fragment of lignin, Fig. 1b is the three phenolic monomers (coniferyl alcohol, coumaryl alcohol, and syringyl alcohol), and Fig. 1c is a structural fragment of phenolic resin. In the molecular structure of lignin the *sp*^2^ aromatic rings of phenol monomers are connected by *sp*^3^ carbon short chains, forming a three dimensional network.

**Figure 2:**
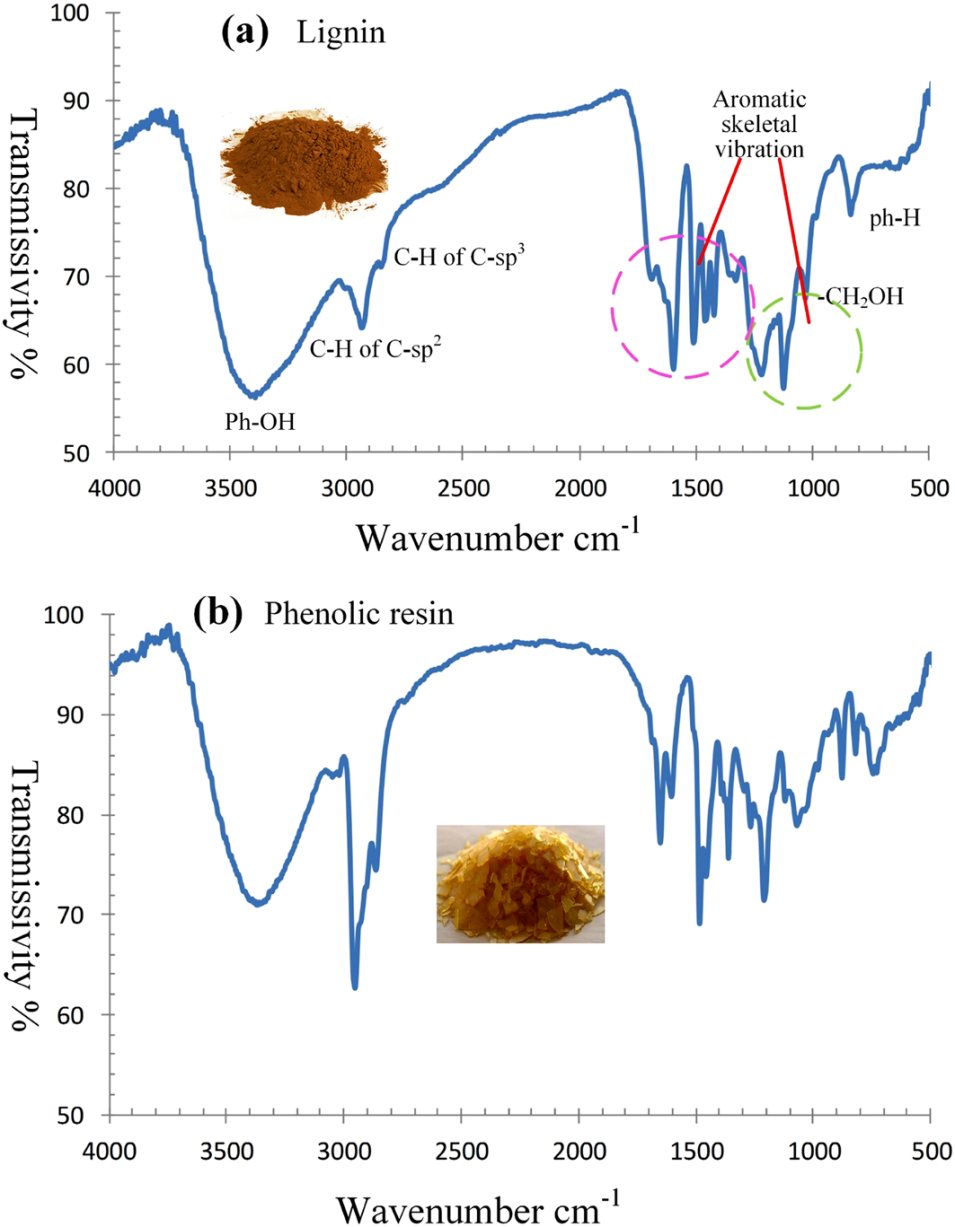
Comparison of FTIR spectrums between lignin and phenolic resin. (**a**) The FTIR spectrum of lignin. (**b**) The FTIR spectrum of phenolic resin. Lignin has several functional groups, alcohol hydroxyl, phenol hydroxyl, carbonyl, and ether group. The FTIR spectrums of lignin and phenolic resin are very similar.

**Figure 3:**
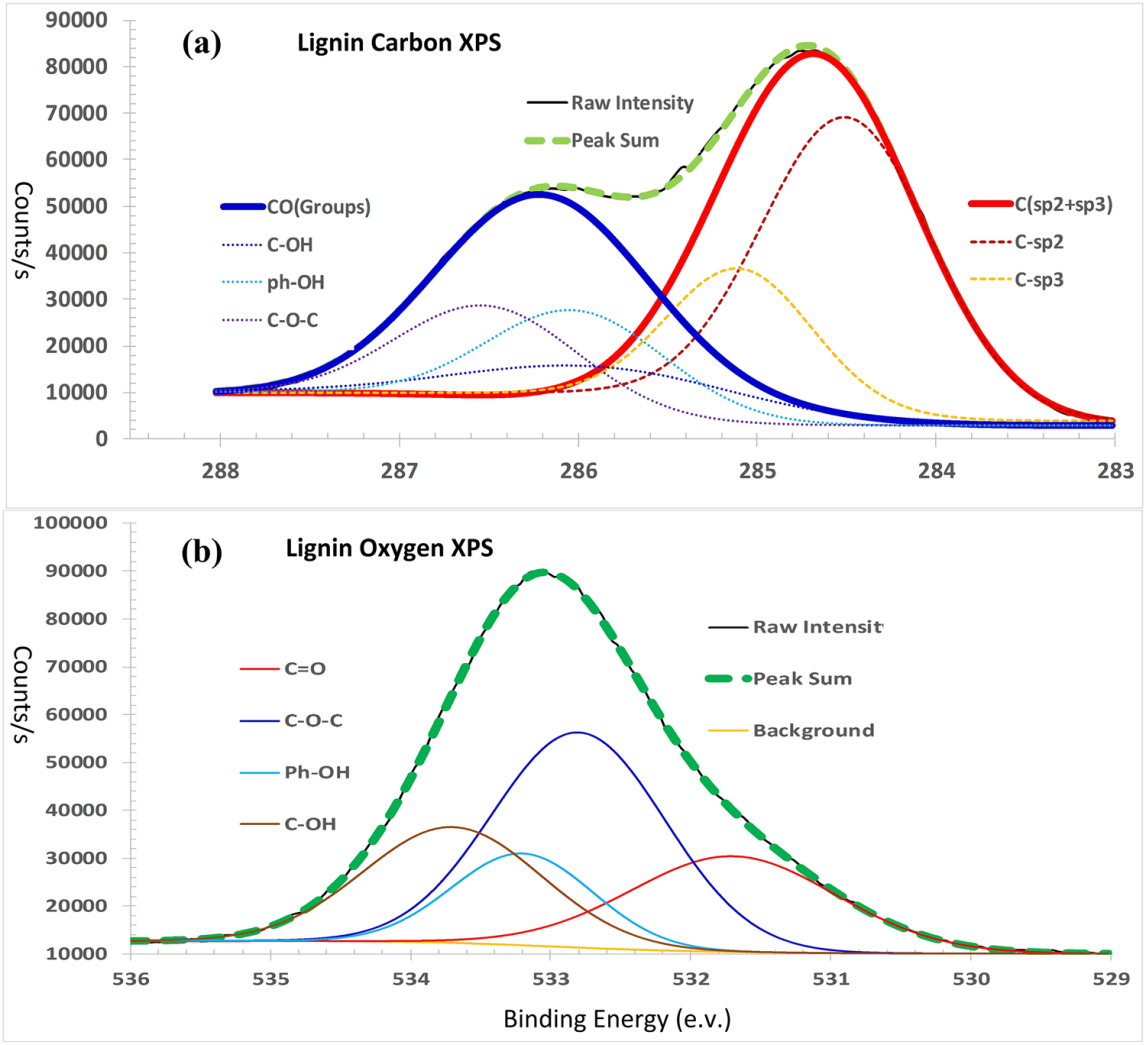
The XPS spectrums of carbon and oxygen in lignin. (**a**) The XPS spectrum of carbon atoms in lignin. There are two overlapped peaks, the red one is the peak of C_1s_ carbon atoms centered at 284.7 eV, composed by two sub-peaks *sp*^2^ (284.5 eV) and *sp*^3^ (285.1 eV) of carbon atoms; and the blue one is the peak of carbon atoms in oxygen groups, centered at 286.2 eV, composed by the sub-peaks of 3 carbon–oxygen groups. (**b**) XPS spectrum of oxygen in lignin. The oxygen XPS spectrum is composed by four oxygen-carbon groups.

Phenolic resin is a widely used precursor for hard carbon materials^26–28^. Comparing lignin with phenolic resin, there are three common grounds: (1) Both are the most carbon rich polymers among all organic polymers, the carbon ratio in lignin is 63.4% and in phenolic resin is 69%. (2) Both are made up of *sp*^2^ carbon aromatic rings and jointed by *sp*^3^ short carbon chains, forming 3D network. (3) Both are random polymers without fixed structures and molecular formulas. However, between lignin and phenolic resin there is a significant difference. Phenolic resin is a stable polymer, in which, except for hydroxyl groups, there are almost no other active functional groups. On the other hand in lignin there are many active functional groups, including alcohol hydroxyl, phenol hydroxyl, carbonyl, ester and ether groups, as indicated in the FTIR spectrum (Fig. 2). In the XPS spectrum of carbon in lignin (Fig. 3a) there are two overlapped peaks between 283 and 288 eV. The red one is the peak of C1s atoms centered at 284.7 eV, composed by the sub-peak of *sp*^2^ carbon (284.5 eV) and the sub-peak of *sp*^3^ carbon (285.1 eV); and the blue one is the peak of carbon atoms in oxygen groups, centered at 286.2 eV, composed by sub-peaks of three carbon–oxygen groups, as listed in Table 1. The XPS peak of oxygen in lignin is composed by four oxygen-carbon groups between 529 and 536 eV, as shown in Fig. 3b. The most favorable aspects of lignin are its very high carbon component (63.4%) and the *sp*^2^ (72.7%) and *sp*^3^ (27.3%) hybrid carbon structure that make it an excellent precursor for *sp*^2^–*sp*^3^ hybrid hard carbon materials, also an excellent substitute for phenolic resin. A remarkable advantage of lignin precursors over phenolic resin is its active functional groups that give lignin new chemical characteristics for development of new carbonization and activation technologies, which will be studied more detailed in the next sections.

**Table 1:** The components of carbon and carbon–oxygen groups in lignin derived from XPS spectrum.

	Atoms	Atomic electron configuration	Position (eV)	Area	Percent
Lignin	C and C in groups	C (*sp*^2^ + *sp*^3^)	284.7	106,937	62.0
		CO groups	286.2	65,413	38.0
Carbon	C atoms	C–*sp*^2^	284.5	74,006	72.7
		C–*sp*^3^	285.1	27,778	27.3
	C in groups	C–OH	285.8	18,351	26.0
		Ph–OH	286.0	26,716	37.8
		C–O–C	286.5	25,582	36.2
Oxygen	O in groups	C=O	531.6	30,993	18.1
		C–O–C	532.6	45,015	26.3
		Ph–OH	533.0	51,253	29.9
		C–OH	533.6	44,222	25.8

#### Recovery of lignin from black liquor

In this section lignin recovery from black liquor by means of acidification^46,47^ and electrodialysis^48,49^ were performed. In pulp black liquor the mass concerntration of dissolved alkali lignin is around 10–18% and mixed by organic and inorganic impurities. The black liquor was first cleaned by using centrifugal filter, and the impurities were removed, including soil, sand, and insoluble organic matters. The filtered black liquor was concentrated to 20% mass percent, and was acidized by 30% H_2_SO_4_ to pH = 2.5, and lignin was precipitated in flocculent precipitate. The suspending lignin was filtered with centrifugal filter, then washed three times with deionized water to neutral. The recovered lignin from black liquor has a higher carbon content than that in biomass due to dehydration reaction during the pulp cooking process^50^. The acidification treatment of black liquor for lignin recovery is a lower cost procedure and could produce in large scale, however, large amount of acid is consumed, and pollutant waste water is produced.

Lignin recovery from black liquor by using electrodialysis (ED) technique^48,49^ was also performed in our research team. After black liquor was filtered using centrifugal filter removing impurities, lignin recovery was carried out in a six compartment ED cell with area 230 cm^2^ and thickness 1.6 cm. The concentration of lignin in mother liquor was increased from 62 to 285 g L^−1^. The suspending lignin precipitate was filtered with centrifugation filter, washed with pH = 2.5 diluted H_2_SO_4_ solution for removing the hemicellulose, then washed three times with deionized water to neutral. Based on our experience the problem of lignin depolymerization during electrodialysis reaction^49,50^ could be solved by aging reaction for a few days. Using electrodialysis approach for recovery of lignin from black liquor, not only can obtain pure lignin, but also can recycle the alkaline solution for pulp cooking, and obtain other useful fractions, such as xylan, in principle no wastes are produced^51^. Due to the remarkable merit, electrodialysis approach has received much attention for cleaner production and utilization of sustainable resource biomass.

#### Preparation of lignin precursors for hard carbon

Lignin has no definite molecular formula and no crystal structure. However, lignin could form various architectures under different pretreatment conditions that affect the structures of carbon products. The lignin recovered from black liquor is further pretreated by using following four methods in authors’ team. (1) In the first method lignin, recovered from black liquor by acidification, was washed using DI water (deionized water) to neutral, then dried in oven at 120 °C for 6 h. (2) In the second method, after black liquor was acidized by 15% H_2_SO_4_ to pH = 2.5, suspending lignin precipitate was washed using DI water, then immediately transferred into a steel beaker, and was rapidly frozen and dried with liquid-nitrogen. (3) In the third method lignin, recovered by electrodialysis, was dissolved in 75% alcohol solution, then solidified using spray-drying technique. (4) In the fourth method lignin was solved in ethylene glycol solution, or *N*,*N*-dimethylformamide solution^52^. Then the lignin membrane was made by using electrospinning technique^53^. Such electrospun lignin membrane, consisting of lignin fiber in micron or nanometer diameter, was heated to the melting point of lignin around 130 °C, and the lignin fiber membrane becomes lignin film in micron or nanometer thickness.

**Figure 4:**
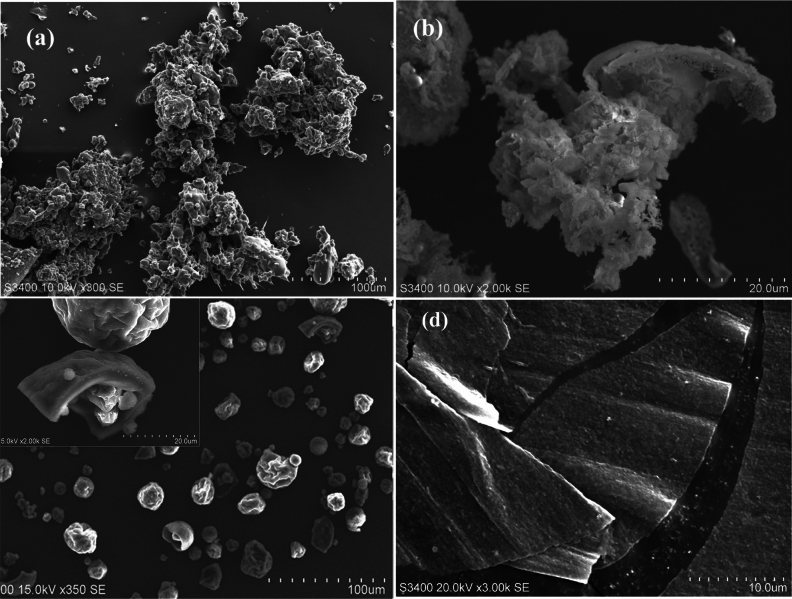
The SEM (scanning electron microscope) images of the lignin particles prepared using four methods. (**a**) The particles of lignin-a, prepared using method 1, are like solid cement slags; (**b**) The particles of lignin-b, prepared using method 2, are like soft cotton; (**c**) The particles of lignin-c, solidified from alcohol solution, are rough balls with diameters from 10 to 20 microns. (**d**) The lignin film-d prepared from lignin-ethanol solution by means of electrospinning technique with thickness around 1 µm.

The SEM images of the lignin precursors, prepared using above four methods, are shown in Fig. 4a–d, labeled as lignin-a, lignin-b, lignin-c and lignin-d, respectively. As shown in Fig. 4a, the particles of lignin-a, prepared using method 1, are like solid cement slags in dimension larger than 100 microns; the particles of lignin-b in Fig. 4b, prepared using method 2, are like softened cotton in dimension around 20 microns; and the particles of lignin-c in Fig. 4c, solidified from alcohol solution, are rough balls with diameters from 10 to 20 microns; and Fig. 4d is the SEM image of lignin film-d prepared using electrospinning technique, and the thickness of lignin film is around 1 µm.

## Results and discussion

### The ***sp***^2^–***sp***^3^ hybrid carbon products from lignin

In this section four hard carbon materials derived from the four lignin precursors are reported and disscused.

#### Lignin derived 3D graphenes

**Figure 5:**
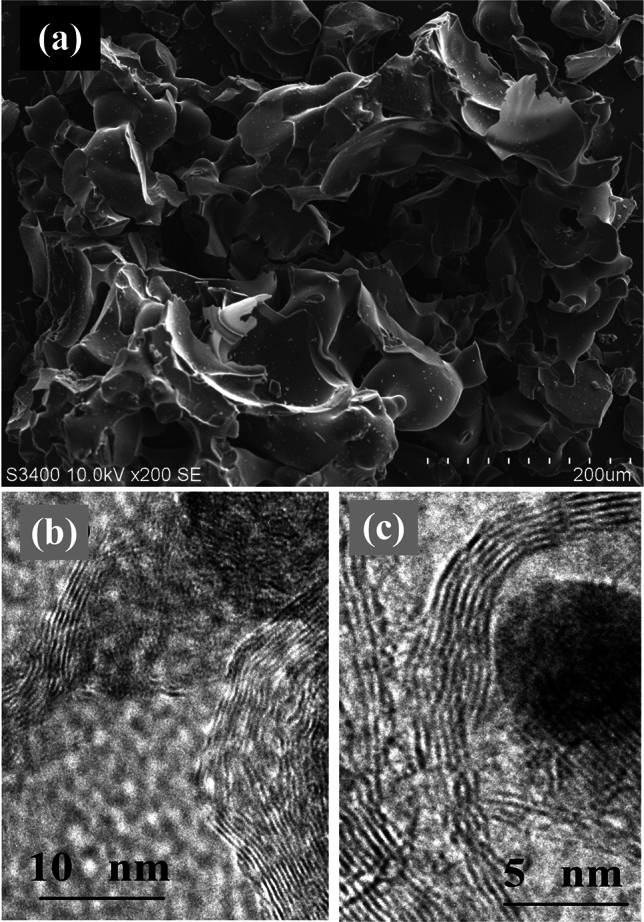
The SEM and HRTEM images of 3D-graphene-b. (**a**) The SEM image of 3D-graphene-b derived from lignin-b. The particles are like variety store: American football, broken earthen jar, and rose petals. (**b**) and (**c**) The HRTEM images of 3D-graphene-b, where the particles of 3D-graphene-b have the fullerene-like carbon crystal structures.

The carbon products, derived from lignin precursors, are strongly impacted by the structures (or architectures) of its precursors. In a published paper of Du’s group^32^ a 3D graphene product was reported that was derived from precursors lignin-a, possessing wood-ear like architecture, is labeled as 3D-graphene-a in this study. In this study a different 3D-graphene is fabricated from precursor lignin-b at 1200 °C for 1 h in nitrogen atmosphere, and is labeled as 3D-graphene-b. Figure 5a is an SEM image of the 3D-graphene-b, in which the 3D graphene particles are like a broken porcelain shop, having different shapes, such as American football, broken earthen jar, and rose petal like sheets, and so on. On the other hand the SEM images of 3D-graphene-a in reference^32^, the 3D graphene particles, fabricated from precursor lignin-a, have wood-ear like architecture. An obvious difference between 3D-graphene-a and 3D-graphene-b is that the curved graphene sheets in 3D-graphene-a merge each other and form dihedral angles with sharp edges; while the graphene sheets of the 3D-graphene-b have smooth and curved surfaces, and have no the edges of dihedral angles, different from those in the former. Figure 5b and c are the HRTEM images of 3D-graphene-b, in which the particles have the graphere and fullerene-like carbon crystal structures^54,55^. It is obvious the architectures of both 3D-graphene-a and 3D-graphene-b are strongly influenced by their precursors lignin-a and lignin-b.

#### Graphene microcrystalline derived from lignin

**Figure 6:**
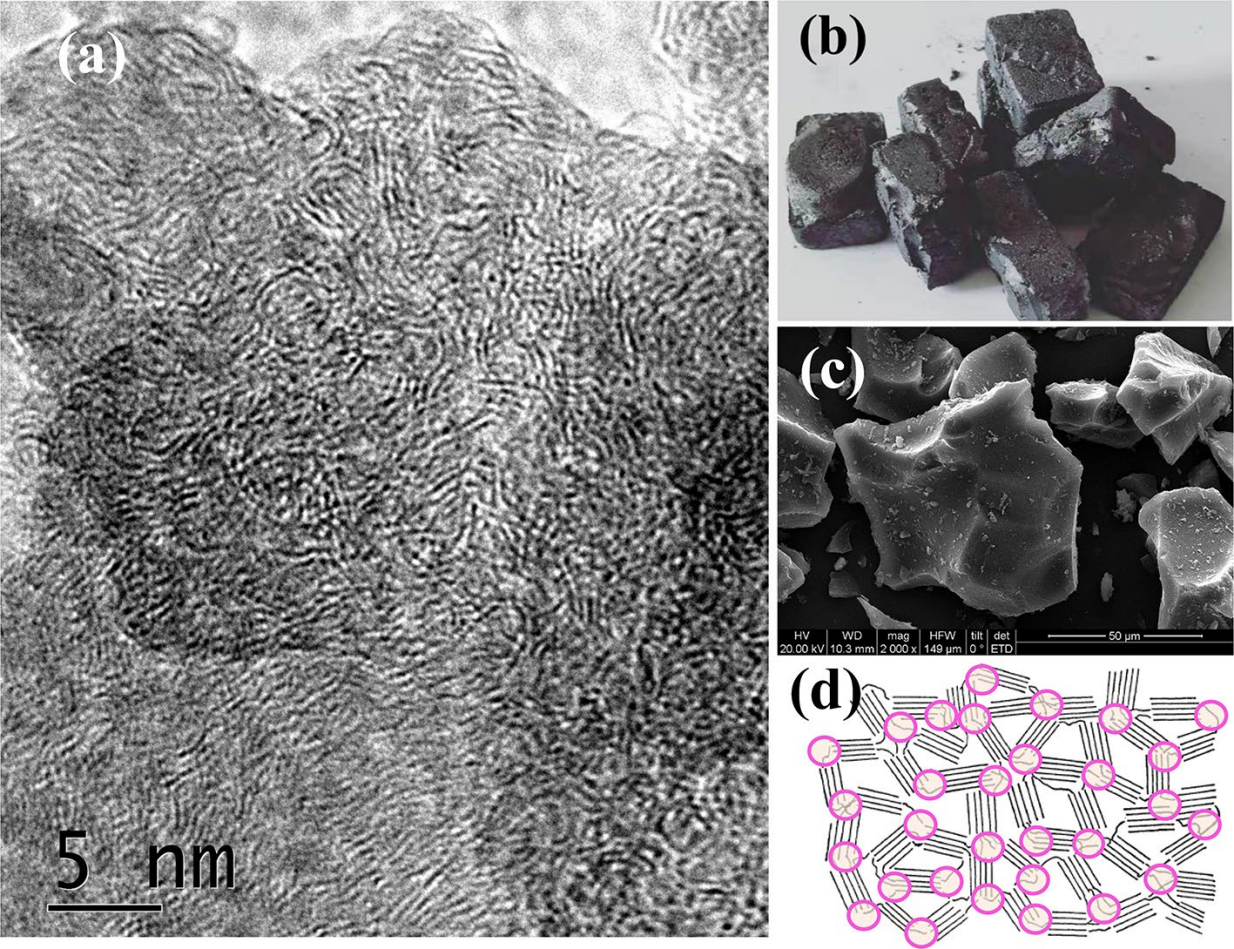
The SEM and HRTEM images of GMC (graphene microcrystalline). (**a**) The 5 nm HRTEM image of GMC. The 3 to 5 parallel lines in length around 3–5 nm are the microcrystals of graphene. (**b**) The photograph of GMC. The hard GMC blocks are like cement slurry. (**c**) The SEM image of GMC. The sharp edges of the GMC particles are very like the glass slags. (**d**) A model structural picture of GMC. The short parallel lines are the microcrystals of graphene, and the join points are the *sp*^3^ carbon atoms, indicated by pink circles.

The graphere microcrystalline (GMC), first reported in reference^35^ of Du’s group, actually is a type of hard carbon^56,57^. In this study GMC was produced from the precursor lignin-c, which was solidified using spray-drying technique from the lignin-ethylene glycol solution. In current investigation the powder of lignin-c was put in graphite crucibles, carbonized in a tube furnace in nitrogen atmosphere at tempereture1200 °C for 1 h, very hard carbon blocks were produced, shown in Fig. 6b and c. The resolution 5 nm HRTEM image of the hard carbon product is shown in Fig. 6a, in which the 3–5 parallel lines in length around 3–5 nm are the microcrystals of graphene. In Fig. 6a the microcrystal units of graphene, arranged in different directions, are jointed by the *sp*^3^ carbon atoms, forming the structure of graphene microcrystalline (GMC). Figure 6c is the SEM image of GMC, where the sharp edges of the GMC particles are very like the glass slags. Figure 6d is a model structural picture of GMC, in which the short parallel lines are the microcrystal units of graphene, and the join points are the *sp*^3^ carbon atoms, indicated by pink circles.

The Brunauer–Emmett–Teller (BET) test was performed for GMC, and the specific surface area (SSA) was almost zero measured using MBET method, and 39 m^2^/g using DFT method, and the total pore volume is merely 0.088 ml/g. All SSA of GMC distribute in the range larger than 5 nm, almost no micro pores, as shown in Fig. 8d. The graphene interlayer spacing of GMC ranges from 0.37 to 0.40 nm^58^, depending on carbonization temperature and lignin precursors, and may be affected by co-reagents. The very hard substrate and the adjustable interlayer spacing of GMC makes it an excellent candidate for anode hard carbon of sodium ion battery^58–61^.

#### Carbon film fabricated from lignin

**Figure 7:**
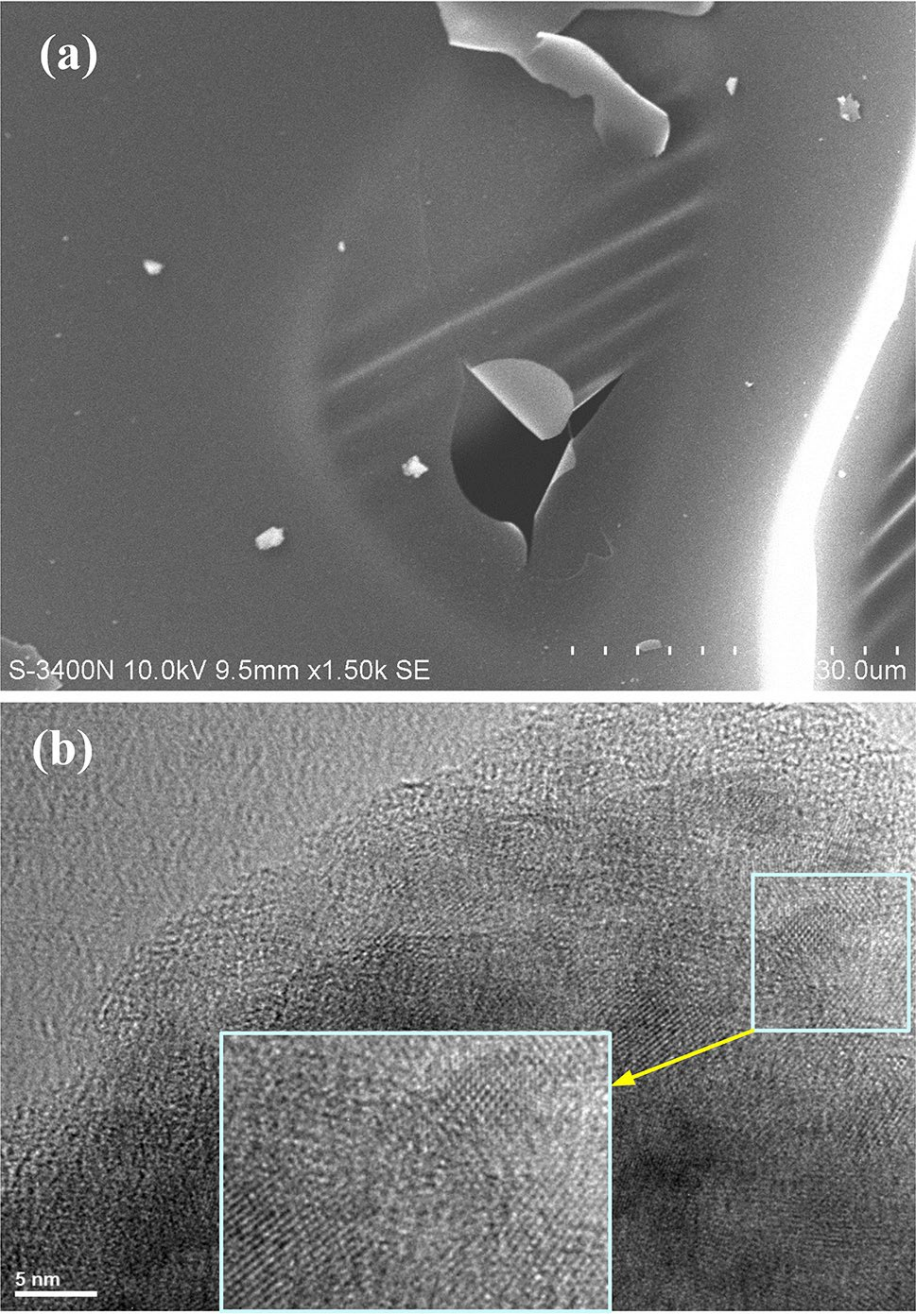
The SEM and HRTEM images of carbon film derived from precursor lignin-d. (**a**) The SEM image of carbon film keeps the film shape of its precursor lignin-d. (**b**) The HRTEM image of the carbon film in 5 nm resolution, consisting of graphene microcrystal units arranged in different directions, where the graphene hexagonal lattices can be seen clearly.

The lignin membrane precursor lignin-d, shown in Fig. 4d, was put in graphite crucibles, carbonized in a tube furnace in nitrogen atmosphere at tempereture1200 °C for 1 h, then carbon films were produced. Figure 7a is a SEM image of the carbon film that keeps the membrane shape of its precursor lignin-d, and Fig. 7b is a HRTEM image of the carbon film in 5 nm resolution, consisting of graphene microcrystal sheets arranged in different directions, where the graphene hexagonal lattices can be seen clearly. In a recent reference^61^ bio-derived hard carbon nanosheets found application in sodium ion battery.

#### Porous carbon fabricated from alkali lignin

**Figure 8:**
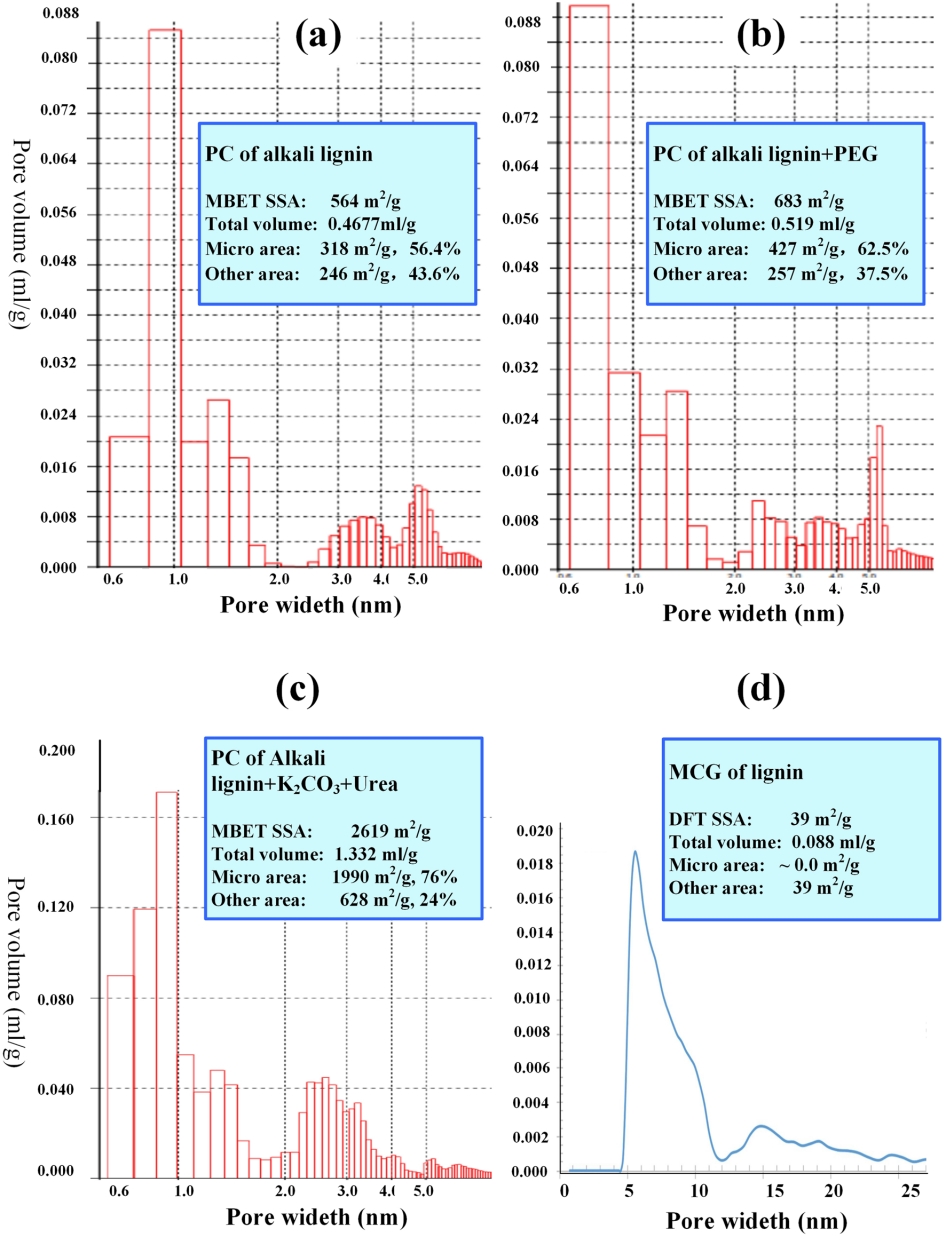
The BET (Brunauer–Emmett–Teller) measurement results of GMC (graphene microcrystalline) and porous carbon derived from lignin. (**a**) The pore volume *vs* pore width of porous carbon of alkali lignin. (**b**) The pore volume *vs* pore width of porous carbon of alkali lignin + PEG-4000. (**c**) The pore volume *vs* pore width of porous carbon of alkali lignin + K_2_CO_3_ + urea. (**d**) The pore volume *vs* pore width of GMC.

The GMC hard carbon, produced from lignin precursor lignin-c in section (2), has very small specific surface area, and almost no micro pore volume smaller than 5 nm. On the other hand, with the help of activating reagents^62^, porous hard carbon could be fabricated from lignin precursors. In this section alkali lignin, provided by Yuanfu Co.Ltd., was used to manufacture porous carbon, in which lignin is the carbon source and alkali is the activating reagent. The powder of alkali lignin was put in graphite crucibles, carbonized and activated in a box-type furnace in nitrogen atmosphere at temperature 750 °C for 1 h. The output porous carbon mixed with molten sodium hydroxide was soaked in clean water, then was filtered for separation of alkaline solution and carbon. The alkaline solution can be directly used for cooking pulp, and porous carbon was washed by 2 M hydrochloric acid, then washed with deionized water to neutral. BET surface adsorption test was performed for porous carbon, and results are shown in Fig. 8a. The BET SSA of porous carbon from alkali lignin is 564 m^2^/g, much larger than that of GMC in Fig. 8d.

In order to promote the micro pores in porous carbon, which is very important for carbon molecular sieves for gas separation, polyethylene glycol (PEG)^63^ was used as an co-activating reagent. Alkali lignin powder was mixed with PEG-4000 in ratio 10:1, then was carbonized and activated using the same methods as above, and the results were shown in Fig. 8b. Comparing with Fig. 8a, after co-activating reagent PEG-4000 was used, the BET SSA, particularly the micro pores smaller that 1 nm, increase remarkably.

The SSA of porous carbon, produced directly from alkali lignin, is not very high, because sodium hydroxide is not a powerful activating agent comparing with expensive potassium hydroxide^64^. However, a remarkable merit is that in the process of making porous carbon form alkali lignin, the alkali consumed by pulping is recovered and can be recycled for pulp production.

In order to produce hard porous carbon with large surface area for supercapacitors, activation reagent potassium carbonate (K_2_CO_3_) and co-activation reagent urea were added in alkali lignin in ratio 1:0.4:0.2. Using the same procedure as above and with the help of K_2_CO_3_ and urea, the SSA of alkali lignin derived hard porous carbon reached 2619 m^2^/g, and the total pore volume was 1.332 ml/g, as shown in Fig. 8c.

### Theoretical analysis of lignin-derived hard carbon

In this section the *sp*^2^–*sp*^3^ hybrid structures of lignin-derived carbon materials, the carbonization mechanism of lignin, and the new activating technique for lignin porous carbon are theoretically analyzed and discussed.

#### The ***sp***^2^–***sp***^3^ hybrid structures

**Figure 9:**
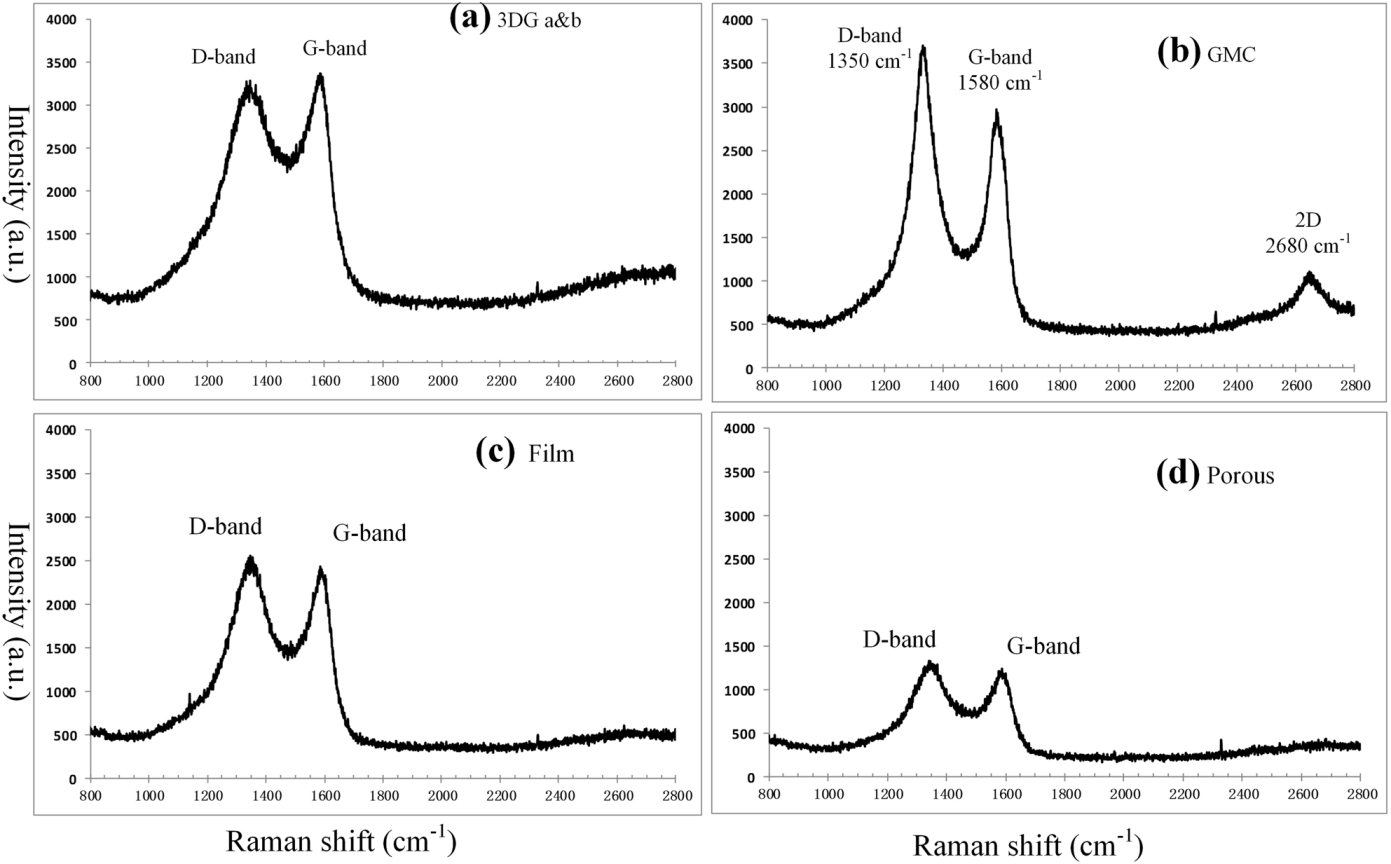
The Raman spectrums of 4 lignin derived hard carbon materials. (**a**) Raman spectrum of 3DG (3D graphene). (**b**) Raman spectrum of GMC (graphene microcrystal). (**c**) Raman spectrum of carbon film. (**d**) Raman spectrum of porous carbon.

The Raman spectrum of typical graphene^65^ has a D-band at 1350 cm^−1^, a G-band at 1580 cm^−1^ and a 2D band at 2680 cm^−1^. The substrate of the four lignin derived hard carbon materials is the graphene microcrystal. Therefore the Raman spectrums of lignin derived hard carbon products are similar to that of graphene, however, disturbed by the *sp*^3^ carbon atoms to different degrees. The Raman spectrums of the four lignin derived hard carbon materials are shown in Fig. 9. In the Raman spectrum of GMC (Fig. 9b) there are all the three Raman bands (D, G and 2D), indicating its finer structure of microcrystal graphene, while other three lignin derived carbon products have no clear 2D-band. Particularly in the Raman spectrum of porous carbon (Fig. 9d) the Raman bands (D and G) are lower than others because of its less finer crystal structure of microcrystal graphene.

**Figure 10:**
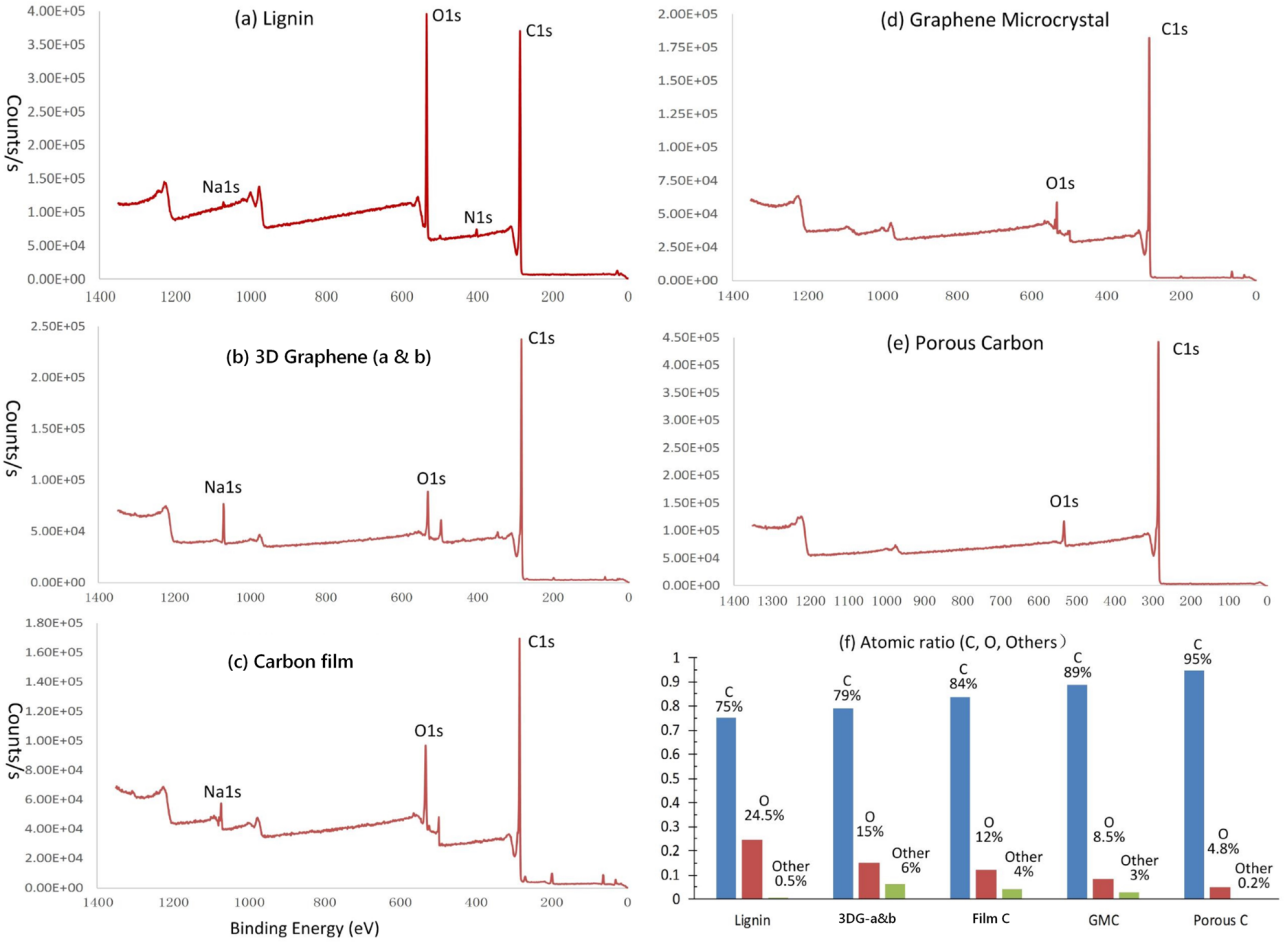
The XPS surveys of lignin and 4 carbon materials derived from lignin precursors. (**a**) XPS survey of carbon in lignin. (**b**) XPS survey of 3D graphene a & b. (**c**) XPS survey of carbon film. (**d**) XPS survey of graphene microcrystal. (**e**) XPS survey of porous carbon. (**e**) The portions of carbon, oxygen and other elements in lignin and four carbon materials.

**Figure 11:**
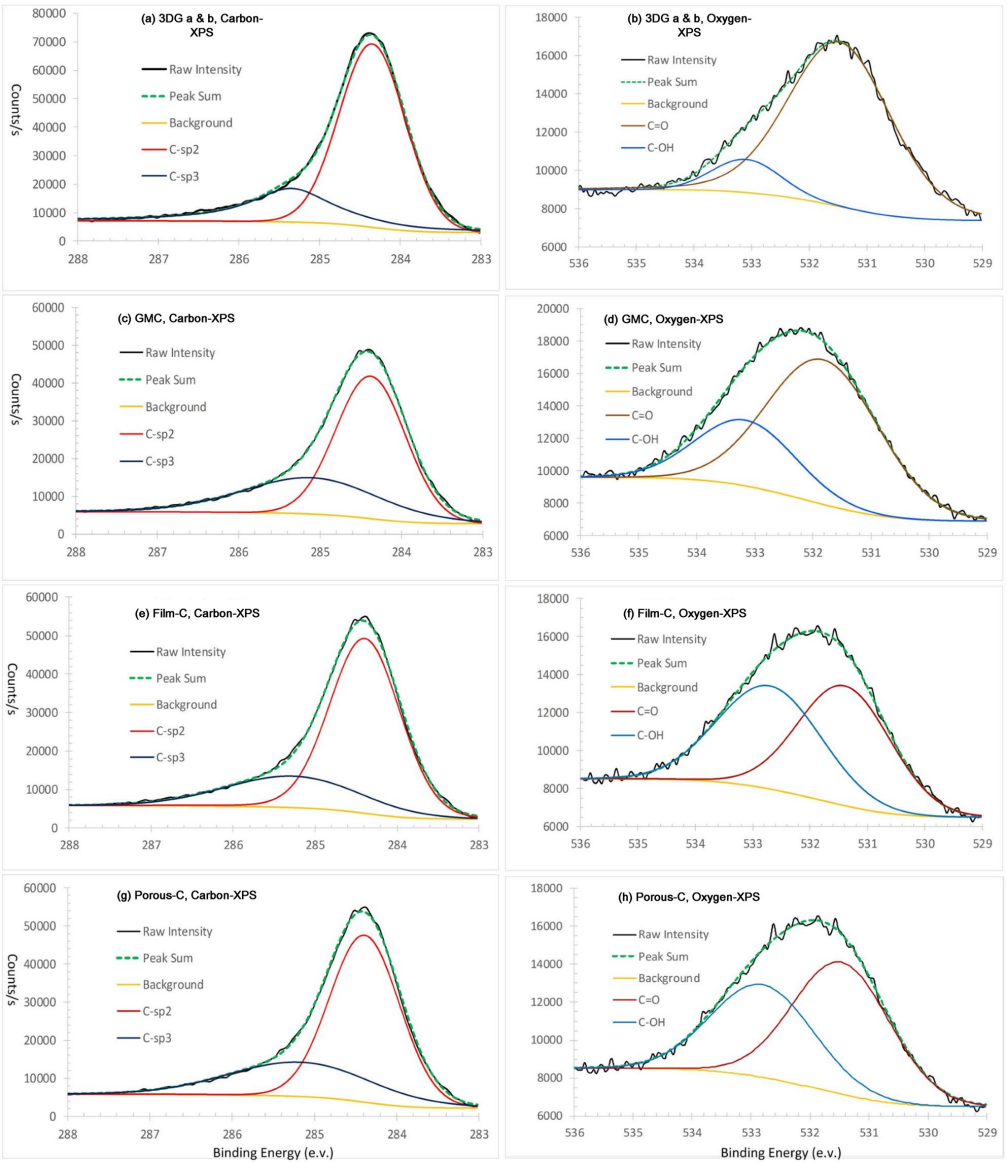
Computational decompositions of XPS spectrums of carbon and oxygen atoms in four carbon materials derived from lignin precursors. (**a**) Carbon XPS of 3D graphene a & b. (**b**) Oxygen XPS of 3D graphene a & b. (**c**) Carbon XPS of graphene microcrystal (GMC). (**d**) Oxygen XPS of graphene microcrystal (GMC). (**e**) Carbon XPS of carbon film. (**f**) Oxygen XPS of carbon film. (**g**) Carbon XPS of porous carbon. (**h**) Oxygen XPS of porous carbon.

The XPS surveys of lignin and 4 lignin-derived hard carbon materials are shown in Fig. 10, where Fig. 10f is the portions of carbon, oxygen and other elements. The peak-fitting technique^66^ was used to separate the overlap peaks of carbon and oxygen in the XPS spectrums of four lignin-derived carbon materials, the results are shown in Fig. 11, and the electron configuration portions of carbon atoms and oxygen atoms are summarized in Table 2.

**Table 2:** The components of carbon and carbon–oxygen groups in lignin-related carbon materials derived from XPS spectrums.

	Atom	Electron configuration	Position (eV)	Area	Percent (%)
3DG-a	C atoms	C–*sp*^2^	284.4	66,219	74.6
		C–*sp*^3^	285.3	22,509	25.4
	O in groups	C=O	531.5	20,549	88.9
		C–OH	533.1	2567	11.1
3DG-b	C atoms	C–*sp*^2^	284.4	40,341	65.2
		C–*sp*^3^	285.1	21,494	34.8
	O in groups	C=O	531.8	21,350	71.2
		C–OH	533.2	8652	28.8
GMC	C atoms	C–*sp*^2^	284.4	50,422	74.8
		C–*sp*^3^	285.2	16,964	25.2
	O in groups	C=O	531.4	12,714	51.0
		C–OH	532.7	12,214	49.0
Porous carbon	C atoms	C–*sp*^2^	284.4	46,751	70.0
		C–*sp*^3^	285.2	20,043	30.0
	O in groups	C=O	531.5	14,359	57.6
		C–OH	532.8	10,569	42.4

Based on Fig. 11 and Table 2 all the 4 lignin-derived hard carbon materials are *sp*^2^–*sp*^3^ hybrid carbon products, and the ratios (65.2–74.8%) of *sp*^2^ carbon atoms and the portions (25.2–34.8%) of *sp*^3^ carbon atoms are close to the ratio in lignin, 72.7% of *sp*^2^ and 27.3% of *sp*^3^, indicating that during carbonization of lignin at higher temperature most carbon atoms keep their original electron configurations.

#### Carbonization mechanism of lignin

A well known phenomenon is that the appearances and architectures of biomass carbon products are very like their biomass precursors, there must be some kind of relationship between bio-precursors and carbon products. The traditional point of view is that carbonization of biomass precursors follows the mechanism “carbonization in situ”^67–70^, it means during the carbonization of biomass organics the carbon atoms stay in their original positions, and the carbon products keep the shapes and architectures of their biomass precursors. In this study the carbonization reactions of lignin precursors follow the same mechanism, and the four hard carbon products keep the architectures of their precursor (lignin-a, b, c, and d). Consequently the architectures and shapes of lignin derived hard carbon materials could be designed by pre-shaping the architectures of lignin precursors.

In this study all the four lignin derived hard carbon products are *sp*^2^–*sp*^3^ hybrid structure, like their lignin precursor. It follows from this phenomenon that in carbonization reaction of lignin, the benzene rings of *sp*^2^ carbon atoms form the graphene fragments, and the *sp*^3^ carbon atoms of short carbon chains joint the nearby graphene units, forming graphene microcrystal units, and most carbon atoms keep their original electron configuration in lignin. Consequently, all carbon products derived from lignin are *sp*^2^–*sp*^3^ hybrid carbon materials with hard graphene microcrystal structures.

#### Activation of lignin derived porous carbon

The traditional production of porous carbon from biomass precursors needs two steps. The first step is the carbonization of precursors, and the second step is the activation of carbon materials using physical or chemical techniques, which are well developed and very familiar for researchers. However, the high carbon ratio and active functional groups in lignin provide the possibility to develop new activation techniques for lignin derived porous carbon production^62^. The activating reagent potassium hydroxide or potassium carbonate can chemically combines with the phenolic hydroxyl groups of lignin, forming alkali-lignin complex, making the activation reaction accurately and efficiently. In addition various co-activating reagents can be used for lignin derived porous carbon, such as urea^62^ and polymer PEG that help pore developing and controlling pore size. A merit of lignin derived porous carbon over other biomass derived carbon products is that the very hard GMC substrate allows lignin porous carbon bearing more holes and larger specific surface area without collapsing than other biomass derived porous carbon products.

## Conclusion

The conclusion points from this study are summarized as follows. (1) The lignin derived hard carbon products consist of microcrystal units of *sp*^2^ graphene fragments, jointed by *sp*^3^ carbon atoms and forming *sp*^2^–*sp*^3^ hybrid hard carbon family. (2) From the lignin precursor to the *sp*^2^–*sp*^3^ hybrid hard carbon products, most carbon atoms retain their original electron configurations (*sp*^2^ or *sp*^3^) and keep their composition in lignin. (3) The architectures of lignin-derived hard carbon materials are closely dependent on the forms of their lignin precursors, and could be preformed by different pretreatment techniques. (4) The carbonization of lignin precursors follows the mechanism “carbonization in situ and recombination nearby”. (5) Due to the high carbon ratio and abundant active functional groups in lignin, new activation techniques could be developed for control of pore size and pore volume. (6) The remaining oxygen atoms in the lignin-derived hard carbon materials are in the forms C=O and C–OH and in the range 5–15%, depending on the carbonization temperature and catalysts, which may provide Faradaic pseudocapacitance and improve hydrophilicity.

In general lignin is an excellent raw material for *sp*^2^–*sp*^3^ hybrid hard carbon products, a green and sustainable alternative resource for phenolic resin. Based on our experiments the carbon yields from lignin to hard carbon products are aroud 40–60%, depending on reaction time and temperature. The yield of lignin-derived porous carbon is 30–40%. Consequently, industrial production for lignin derived hard carbon materials would be feasible, also beneficial to ecological and environmental protection, and also to sustainable economy.

## Data Availability

All data that support the findings in this study are in the article, and asking for detailed experimental data is possible depending on further communication.
